# Swimming against the currents: Our experience providing primary healthcare services for the stateless community in Semporna

**DOI:** 10.51866/mol.571

**Published:** 2024-04-13

**Authors:** Mei Yee How, Shu Hui Ng

**Affiliations:** 1 MD, MFamMed, Klinik Kesihatan Apas Balung, Jalan Apas Balung, Tawau, Sabah, Malaysia. Email: meiyeehow29@gmail.com; 2 MBBS, MRCP, LL.M, Angsana Health, BO1-A-9, Menara 2, KL Eco City, 3, Jln Bangsar, 59200 Kuala Lumpur WP, Malaysia.

**Keywords:** Stateless community, Medical outreach programme, Primary healthcare services

## Background

Malaysia provides robust universal health coverage for its citizens, and primary healthcare services are easily accessible to most Malaysians.^1^ However, access to healthcare does not extend to the stateless population, including the Bajau Laut community in Semporna, East Sabah. Semporna is often touted as Asia’s best aquatic paradise, with divers and tourists from across the world flocking to the region to experience the spectacular waters and diverse marine life. Many people remain oblivious to the challenges endured by the stateless population, including the lack of access to basic primary healthcare.

The Bajau Laut’s presence and freedom of movement within their ancestral domain in the Sulu and Celebes Seas or the Sulu–Semporna–South Sulawesi Triangle have been well-established since the pre-British era.^2^ Challenges have arisen regarding their legal standing as the formalisation of citizenship and territories has occurred with the emergence of the nation-states of the Philippines, Malaysia and Indonesia. In Sabah, there are three groups of Bajau: The West Coast Bajau, the various groups of East Coast Bajau and the Bajau Laut residing along Sabah’s east coast and surrounding islands near Semporna. The West Coast and East Coast Bajau have gradually assimilated into Malaysian society, whereas the Bajau Laut maintains a semi-nomadic maritime lifestyle and is typically considered stateless by the Malaysian government.^2^

Besides severely restricted access to universal health coverage, the stateless community in Malaysia is also excluded from basic education and employment opportunities.^3^ As a result, it is trapped in a vicious cycle of statelessness, illiteracy and poor health, with little to no means of breaking out from intergenerational poverty. Deemed as non-citizens or illegal immigrants, the stateless community is often ‘invisible’, and many of their struggles remain ‘unseen’ and ‘unheard’.

## Overcoming barriers

Owing to significant constraints in accessing the public healthcare system, most stateless children are delivered at home and receive no childhood immunisation or any form of healthcare. Based on our anecdotal experience and corroboration with community members, children who do not survive childhood are often buried by their families, with no birth or death statistics recorded. Visitors to Semporna will be familiar with stateless children who often roam the streets of the town. Most of these children are illiterate, with no chance for school or any form of employment. They sell foraged seafood or plastic bags to tourists, earning a mere MYR 4–6 (~USD 1) per day to support their families. Even more heart-wrenchingly, children have to resort to begging for money or food just to survive. As a result, they suffer from malnutrition, head lice and scabies infestations from poor sanitation and various soft tissue injuries or infections from prolonged hours of walking without proper footwear.

Recognising the tremendous need for healthcare among the stateless population in Semporna, we set up a voluntary medical outreach programme managed by a small team of four doctors and four pharmacists and hosted at a private guest lodge off Pulau Bum Bum. The outreach programme aims to bridge the healthcare gap in both acute and chronic diseases for all ages among the stateless and underprivileged community. We named our service ‘Sunset Clinic’ because our programme extends through sunset to cater to the Bajau Laut fishermen’s schedule.

The programme is organised on a quarterly basis to ensure continuity of care, with basic health equipment and medications funded by both private and corporate donors. Over the past year, we have contacted local community leaders and village heads to inform them about any upcoming medical outreach events. This has helped us reach those in need of medical care, either via invitations to our clinic or via word-of-mouth within the community. Our partnering host makes logistics arrangements, coordinating effectively with community members so that patients could reach Sunset Clinic by boat.

We have gradually gained more trust from the stateless community and have since treated more than 300 patients aged from 8 months to 80 years. The medical conditions we commonly encounter are communicable diseases such as scabies, hair lice and worm infestations as well as acute respiratory and gastrointestinal infections and non-communicable diseases such as chronic untreated asthma, hypertension, diabetes and childhood malnutrition. We practise the foundational family medicine concept in our medical outreach programme by providing personalised and comprehensive care to those in need. The family doctors’ expertise in all areas of healthcare, including non-communicable diseases, infectious diseases, maternal and child health and preventive care, is pivotal in addressing the diverse healthcare challenges in this resource-limited environment. The family doctors have helped to not only manage a wide range of acute to chronic health issues but also incorporate a deep understanding of how environmental, social and economic factors can impact the overall well-being of individuals, their families and the community.

In the past year, we have conducted basic health screenings, outpatient medical services and much-needed dental services for the stateless community through collaborative work with other non-governmental organisations. Most of our patients have poor oral health and dental hygiene. Tooth extractions and cavity fillings are performed on most of them. During these events, we also take every opportunity to provide basic health and dental education. Children especially enjoy these events, as they are usually provided with toothbrushes and toothpastes and get to eat a full meal during their time with us. Sadly, for the stateless children in Semporna, having a full meal and even eating with cutleries are considered a luxury.

Due to logistical and financial barriers, our clinic runs with the most basic medical equipment, and we have yet to be able to perform diagnostic laboratory examinations. Our team relies heavily on comprehensive history-taking and physical examination for diagnosis. Irregular funding mechanisms also mean that we cannot supply long-term medications for prolonged periods. We seek funding periodically through donation drives or engagements with corporate entities for medication sponsorship. Patients who come with more serious medical conditions receive a referral letter to a governmental clinic nearby or to the tertiary hospital in Tawau. Financial constraints are inevitable, but we do as much as we can to facilitate care within our means and capacity.

## Our hope for the future

Our team is fully cognisant that we cannot (or will never be able to) exert immediate change to social determinants of health, such as the provision of clean water, sanitation of the environment and improvement of the socioeconomic status. However, we strongly believe in a multiprong approach involving education, direct provision of healthcare services and advocacy for systemic change in achieving improved health outcomes for the stateless population. Systemic change requires collective commitment from policymakers and all healthcare professionals in the implementation of inclusive policies to support the attainment of health and education for all.

In particular, we recognise the absolute importance of education for the stateless children in Semporna. Therefore, we aim to collaborate with local alternative schools to strengthen health education and support access to basic education for these children. Education is crucial to empower a generation to seek change for themselves. Sunset Clinic also envisions strengthening existing services and expanding healthcare services to include the provision of childhood immunisation to the stateless children.

Maternal care is also crucial, but we recognise our limitation of not being able to provide basic obstetric services, as we do not have the human and financial resources to provide comprehensive and continuous care for pregnant women. However, knowing the importance of maternal care in reducing maternal and perinatal mortalities, our team hopes to collaborate with local family physicians or general practitioners to ensure the sustainability of maternal care initiatives.

For the older adult population, we have recognised logistical barriers in reaching our clinic. They often face difficulties in boarding and disembarking from boats as well as in navigating steep and slippery wooden stairs at the jetty. To address this issue, we are actively exploring alternative venues to host Sunset Clinic, such as an accessible community hall, and organising home visits to improve healthcare access for the older adult population.

We hope that this article will implore every Malaysian and visitor to Semporna to seek a deeper understanding of the issue of statelessness and how it impacts the health and lives of the stateless population in Sabah. Although we often receive feedback and comments from the local community and colleagues from the medical fraternity that our work is a mere ‘band-aid’ solution to a massive public health and systemic citizenship issue, our team is convicted that health is a human right and that no one should be completely forsaken, regardless of their citizenship. Through increased awareness, coordinated efforts and deeper engagement with all stakeholders in health and education, we hope to positively impact every life we touch, regardless of the scale of such impact.

**Figure 1:**
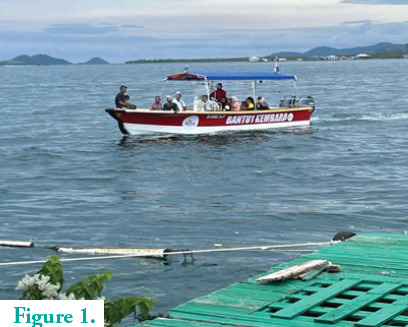
Villagers arriving by boat.

**Figure 2:**
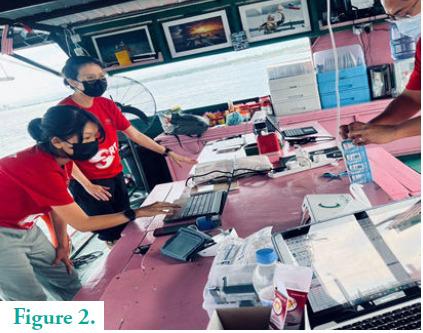
Preparation before the start of the clinic session.

**Figure 3:**
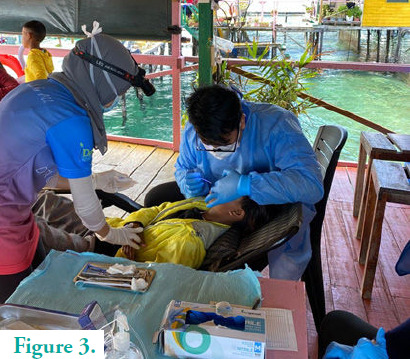
Dental team performing tooth extraction.
